# Warfarin Sodium Stability in Oral Formulations

**DOI:** 10.3390/molecules26216631

**Published:** 2021-11-01

**Authors:** Evangelia Dimitrokalli, Stefani Fertaki, Michail Lykouras, Petros Kokkinos, Malvina Orkoula, Christos Kontoyannis

**Affiliations:** 1 Department of Pharmacy, University of Patras, GR-26504 Rio Achaias, Greece; evadim@hotmail.com (E.D.); sfertaki@gmail.com (S.F.); michalislyk@gmail.com (M.L.); malbie@upatras.gr (M.O.); 2 Institute of Chemical Engineering Sciences, Foundation of Research and Technology-Hellas (ICE-HT/FORTH), GR-26504 Platani Achaias, Greece; pkokkin@upatras.gr

**Keywords:** warfarin sodium amorphous, warfarin, warfarin clathrate, oral suspension, crystallization, Raman spectroscopy, X-ray powder diffraction, DSC, IR

## Abstract

Warfarin sodium is a low-dose pharmaceutical blood thinner that exists in two forms: the clathrate form and the amorphous form. In commercially available warfarin sodium oral suspension, the active pharmaceutical ingredient (API) is added in the amorphous state. This study investigates the apparent instability of the commercially available warfarin liquid oral formulation using Raman and IR spectroscopy, X-ray diffraction, differential scanning calorimetry, UV spectroscopy, and optical microscopy. Warfarin, not its sodium salt, was identified as the undissolved solid existing in the suspension. This was found to be due to the dissociation of sodium salt and the protonation of the warfarin ion in the liquid phase, which triggered the crystallization of the sparingly soluble unsalted form. The coexistence of protonated and unprotonated warfarin ions in the supernatant, as detected by Raman and UV spectroscopy, confirmed this assumption. Study of the dissolution of warfarin sodium amorphous salt and crystalline sodium clathrate in the placebo and pure water verified the results. The effect of pH and temperature on warfarin precipitation was also explored.

## 1. Introduction

Polymorphism can be defined as the ability of a drug compound to crystallize into more than one crystalline form that differs in molecular packing arrangements and/or conformations within the crystal lattice. It has been widely reported that pharmaceutical solids can exist in more than one form under certain conditions. The different polymorphs are usually characterized by different physical and chemical properties, such as stability, solubility and, occasionally, bioavailability. On the other hand, active pharmaceutical ingredients (APIs) may also be used in pharmacy in the amorphous state, which lack crystallinity. They, usually, exhibit higher solubility, higher dissolution rate, and better compression characteristics than the corresponding crystals [1]. Their inherent instability may affect their bioavailability. For this, care must be taken when amorphous APIs are used.

Any changes in the structure or crystallinity of the APIs should be taken into consideration in order to control the properties of the APIs and final products. It is a common requirement in pharmacopeia monographs that APIs in drugs exist in a single fixed crystalline form [2,3]. The application of numerous analytical techniques has been reported in the identification of polymorphs, including Fourier transform infrared spectroscopy (FTIR), Raman spectroscopy, solid-state nuclear magnetic resonance spectroscopy (ssNMR), thermal methods, and X-ray powder diffraction (XRPD) [2].

Warfarin is one of the most common oral anticoagulants used to treat venous thrombosis and pulmonary embolism, prevent prosthetic heart valve thromboembolism, and reduce the risk of death by recurrent myocardial infarction and thromboembolic events such as stroke and systemic embolization following myocardial infarction [4].

Warfarin is very soluble in alkaline solutions and produces salts, the most well known of which is warfarin sodium. The latter exists in two solid forms: the amorphous form and the isopropyl alcohol solvate crystalline form. The amorphous form is stable under ambient conditions. The crystalline form is a warfarin sodium–isopropyl alcohol complex, known as warfarin clathrate [5]. It is prepared from either warfarin or amorphous warfarin sodium in order to eliminate impurities in warfarin sodium [6,7]. The isopropyl alcohol and water molecules play a critical role in the formation and stability of the solvate, since the warfarin sodium/isopropyl alcohol/water ratio may vary from 8:4:0 to 8:2:2 and still possess a monoclinic space lattice [7,8]. Hiskey and Melnitchenko (1965) focused on the continuous series of compositions that result, and reported that the crystallinity of the clathrate form is reduced with an increase in water content [8].

**Figure 1:**
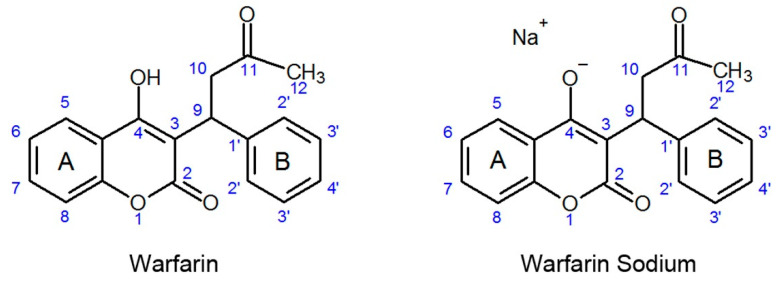
The molecules of warfarin [12] and warfarin sodium [13]. The OH group at C4 atom of warfarin is deprotonated to form warfarin sodium.

The molecular and crystal structure and physical properties of warfarin sodium crystalline clathrate have been extensively investigated [7,8,9,10]. Less information is available regarding the amorphous state, e.g., data regarding solubility are scarce. When contained in tablets, both the warfarin sodium amorphous and warfarin sodium crystalline clathrate forms are converted to a crystalline protonated form (Figure 1) in the acidic pH of the stomach, as was revealed by XRD and Raman spectroscopy. The chemical properties of the new protonated form were shown to be independent of the initial form of warfarin sodium API [11].

As a result of the fact that warfarin is practically insoluble in aqueous solutions, the salt of warfarin sodium was used as an API in the first formulation, Coumadin^®^, a tablet form from Bristol Myers Squibb in the United States. Coumadin^®^ is available in different strengths, ranging from 1 to 10 mg. Many generic products are currently available worldwide, e.g., Jantoven^®^. Nowadays, the drug is also available as lyophilized powder for injection and oral suspension. In 2010, Rosemont Pharmaceuticals launched a new oral suspension, warfarin sodium product (with a strength of 1 mg/mL), which was the first of its kind in the United Kingdom. The new product aimed to make dose adjustment more precise and therefore increase the safety of the drug for patients, as compared to tablets. Furthermore, it aimed to be more suitable for infants and patients over 60 years old who have difficulty swallowing warfarin tablets and other solid-dose medications, and those with transient swallowing difficulties, e.g., patients who have had a stroke [14,15]. Although the crystalline clathrate polymorph of warfarin sodium is usually added in the tablet, for the oral suspension, warfarin sodium is added in the amorphous state as it was considered to be more suitable for the liquid oral formulation due to its increased solubility. However, the final product appeared as a suspension and not as a solution.

In the present work, the apparent instability of the commercially available warfarin liquid oral formulation was investigated. The chemical identification of the precipitate triggered an in-depth study of the soluble forms of warfarin in aqueous solutions. The established analytical technique for warfarin determination is HPLC [5,16]. Here, spectroscopic techniques (Raman, XRPD, IR, and UV), optical microscopy, and thermal (DSC) analytical techniques were employed for the characterization of the produced solids and the remaining saturated liquid phases. The effect of pH and temperature on the crystallization of the API were also investigated.

## 2. Results

### 2.1. Raman Spectroscopy of Warfarin Solid Forms, Warfarin Oral Suspension, and Warfarin Solutions

**Figure 2:**
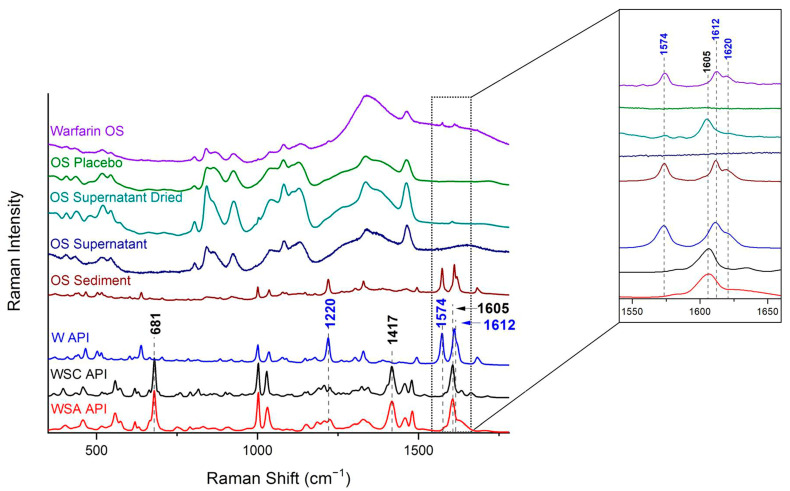
Raman spectra of the warfarin oral suspension (OS), oral suspension placebo, oral suspension supernatant dried, oral suspension supernatant, oral suspension sediment, warfarin (W) API, warfarin sodium clathrate (WSC) API, and warfarin sodium amorphous (WSA) API. Characteristic peaks of the APIs are indicated. Insert: Magnification of spectral area 1550–1650 cm^−1^.

Warfarin and the two sodium salts were studied with Raman spectroscopy. Their spectra were recorded (Figure 2) and found to be in good agreement with the literature [11]. They were used henceforth as references. As can be seen, the spectra of the two sodium salts are quite similar, and differentiation between them is difficult. Conversely, warfarin exhibits distinct peaks for discrimination against the salts: the double peak at 1574 cm^−1^ (assigned to the C-C stretching vibration 8b mode of the phenyl B group) and 1612 cm^−1^ (attributed to the C-C stretching vibration 8b mode of the phenyl A group, coupled with the C-H bending mode), and the peak at 1220 cm^−1^ (O-H in-plane bending) [17].

The commercially available oral suspension was studied as received and after centrifugation and separation of the liquid phase (supernatant) and the solid (sediment). Their Raman spectra are also presented in Figure 2.

In the spectrum of the oral suspension, despite its domination by placebo peaks, the presence of warfarin is evident (double peaks at 1574 and 1612 cm^−1^). Contrary to what was expected, the sodium salt was not detected, since there was an absence of any of the expected peaks at 681 cm^−1^ (out-of-plane skeletal mode 4 of phenyl B group), 1417 cm^−1^ (CH_2_ rocking bond) and 1605 cm^−1^ (C-C stretching vibration 8b mode of the phenyl A group) [18]. Furthermore, the spectrum of the sediment removed by centrifugation revealed exclusive precipitation of the unsalted form of warfarin, which was identical to that of the reference (Figure 2). No warfarin could be detected in the supernatant, probably due to its limited concentration in the liquid phase. On the other hand, the initial addition of the sodium salt in the formulation was verified (together with warfarin; peak at 1574 cm^−1^) in the solid that precipitated after a small quantity of the supernatant was quickly dried in an oven.

It can thus be concluded that the warfarin sodium salt in the formulation is not stable. Precipitation of the warfarin takes place, and the solution becomes a suspension. A possible explanation for this is that the warfarin ion from sodium salt dissolution becomes protonated in the acidic pH of the formulation and subsequently precipitates due to low solubility.

**Figure 3:**
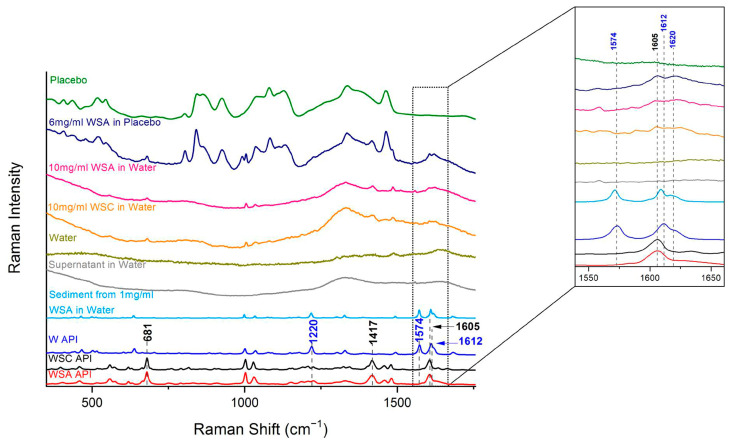
Raman spectra of warfarin placebo, warfarin sodium amorphous (WSA) solution in placebo (6 mg/mL), warfarin sodium amorphous solution in water (10 mg/mL), warfarin sodium clathrate (WSC) solution in water (10 mg/mL), water, supernatant of WSA in water (10 mg/mL), sediment of the suspension of 10 mg/mL WSA in water, warfarin (W) API, warfarin sodium clathrate (WSC) API, and warfarin sodium amorphous (WSA) API. Characteristic peaks of the APIs are indicated. Insert: Magnification of spectral area 1550–1650 cm^−1^.

To verify the previous result, warfarin sodium amorphous salt was dissolved in the placebo solution and in water at concentrations 6 and 10 mg/mL, respectively. Dissolution occurred readily, i.e., clear liquids were prepared. Raman spectra, presented in Figure 3, reveal the transformation, though incomplete, of the sodium salt into warfarin in the liquid phase (peaks at 1605 and 1620 cm^−1^, respectively) in accordance with the assumption made previously for the oral suspension. Soon after, the solutions turned cloudy. Raman spectra of the sediments and the supernatants separated by centrifugation were collected (Figure 3). Warfarin was identified in each of the two sediments, in agreement with the findings from the oral suspension. No warfarin could be detected in the supernatant spectra, as before.

Dissolution of sodium clathrate salt in water (1 mg/mL) was also tested. The findings were similar (Figure 3).

### 2.2. X-ray Diffraction of Warfarin Solid Forms and Sediments

**Figure 4:**
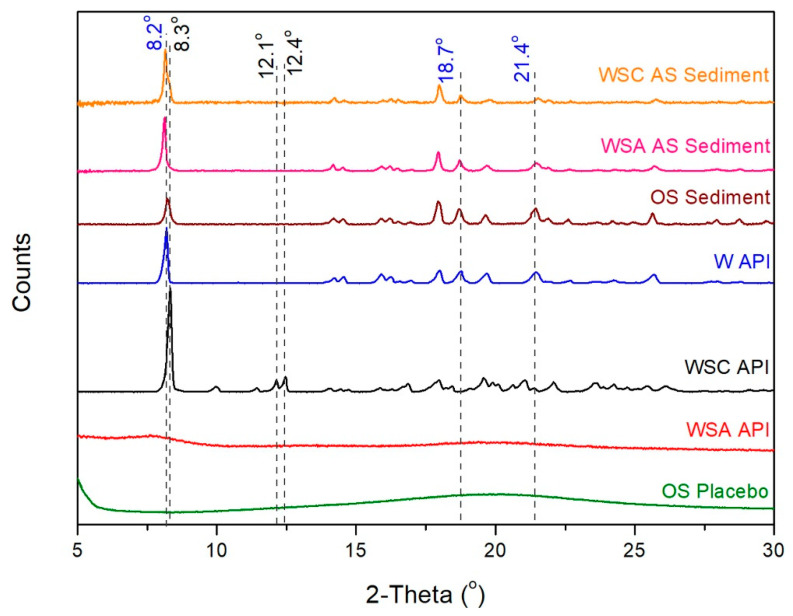
XRPD patterns of the sediment obtained from the aqueous solution of warfarin sodium clathrate (WSC AS), the sediment obtained from the aqueous solution of warfarin sodium amorphous (WSA AS), oral suspension (OS) sediment, warfarin (W) API, warfarin sodium clathrate API, warfarin sodium amorphous API, and oral suspension placebo.

The sediments from the previous experiments (Section 2.1) were further investigated using X-ray powder diffraction. All diffractograms recorded can be seen in Figure 4.

The three APIs were also scanned, and their diffractograms were used as references. The warfarin sodium amorphous salt exhibits no peaks, as was expected. The crystalline APIs can be easily distinguished. Apart from a minor shift of peak at 8.2°, the peaks at 18.7° and 21.4° demonstrate the presence of warfarin. The spectra are in good agreement with the literature [11,19].

From the results, it is apparent that all sediments consist of warfarin, as was also evidenced by Raman spectroscopy.

### 2.3. IR Spectroscopy of Warfarin Forms and Sediments

**Figure 5:**
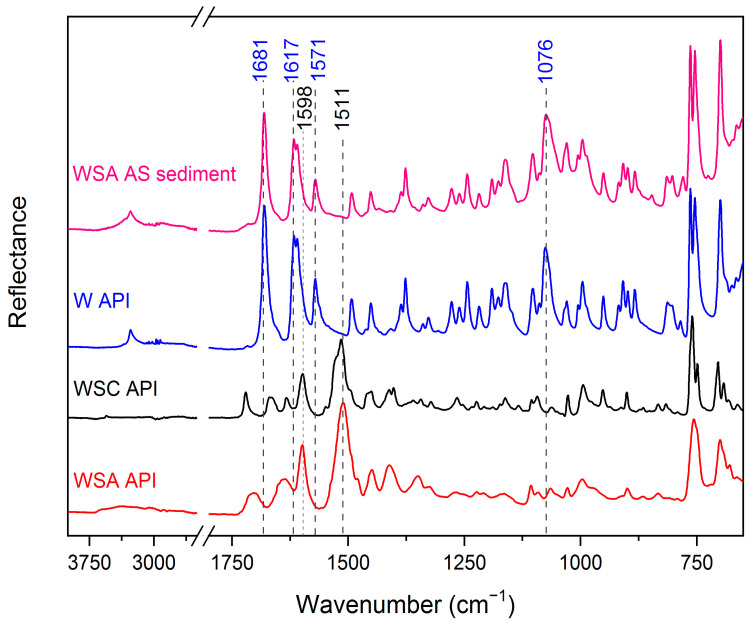
ATR spectra of the sediment obtained from the aqueous solution of warfarin sodium amorphous (WSA AS), warfarin (W) API, warfarin sodium clathrate (WSC) API, and warfarin sodium amorphous API.

The IR/ATR spectra of the sediments verify the presence of warfarin, while neither of the salts (amorphous or clathrate) could be detected (Figure 5). The absence of peaks at 1598 cm^−1^ (C-C stretching vibration 8b mode of phenyl A group) and 1511 cm^−1^ (C-C stretching vibration 8a mode of phenyl B group) [18] proves that neither warfarin sodium amorphous nor warfarin sodium clathrate are present in the sediment. On the other hand, the peaks of 1681 cm^−1^ (stretching of the carbonyl group of C_11_ atom), 1617 cm^−1^ (C-C stretching vibration 8b mode of phenyl A group, coupled with the CH bending mode), 1571 cm^−1^ (C-C stretching vibration 8a mode of phenyl B group), and 1076 cm^−1^ (C-H in-plane bending vibration 18a mode of phenyl B group) [17,20] in the sediment acquired by the aqueous solution of warfarin sodium amorphous and in warfarin API spectra verify that they are chemically identical.

### 2.4. DSC Analysis of Warfarin Forms and Sediments

**Figure 6:**
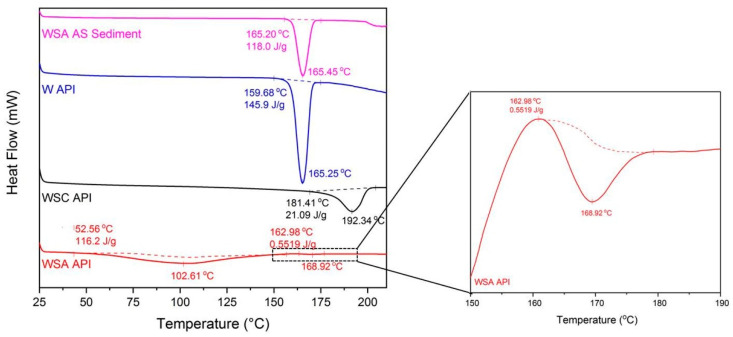
DSC thermograms of the sediment obtained from the aqueous solution of warfarin sodium amorphous (WSA AS), warfarin (W) API, warfarin sodium clathrate (WSC) API, and warfarin sodium amorphous API. Insert: Magnification of the warfarin sodium amorphous API thermogram in the range of 150–190 °C.

Of all the techniques used in the present work, differential scanning calorimetry appears to be the only one that can discriminate between the three forms of warfarin (Figure 6). The sharp endothermic curve that emerged at 160.2 °C (peak at 165.45 °C) corresponds to the melting point of warfarin, which is in agreement with the literature [6]; whereas for warfarin sodium in amorphous form, a broad endotherm at approximately 100 °C and a small endotherm at approximately 160–165 °C were observed. The broad endotherm corresponds to humidity loss, also verified by a second heating cycle. Due to lack of the periodic structure in an amorphous system, a significant adsorption of water is expected [7]. The small peak noticed at 160–170 °C is the glass transition temperature of warfarin sodium amorphous (insert of Figure 6), confirmed by visual inspection of a sample dried in the lab oven, as well. For warfarin sodium clathrate API, a distinct endothermic peak at 192.34 °C was detected, which corresponds to the evolution of isopropanol and the subsequent melting of the warfarin sodium crystalline form [7].

Therefore, using DSC, it can be concluded that the sediments comprise solely warfarin. This is in agreement with the assumption that warfarin sodium salt is transformed to a protonated warfarin ion, which immediately precipitates.

### 2.5. Crystal Morphology of Warfarin Forms and Sediments

**Figure 7:**
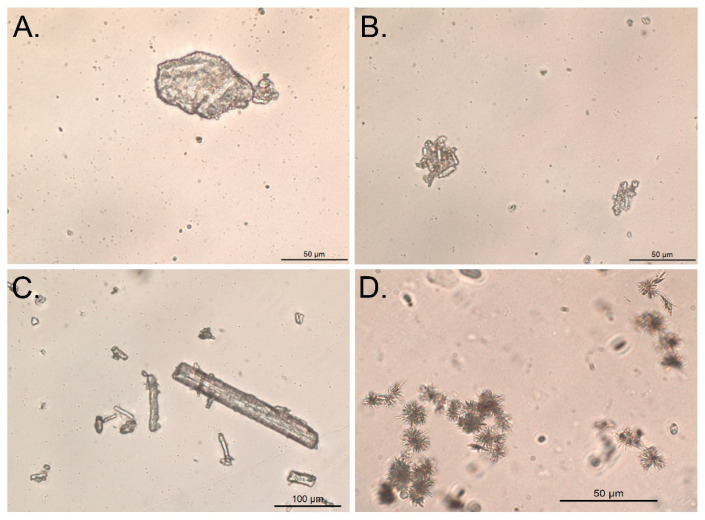
Optic microphotographs of warfarin sodium amorphous API (**A**), warfarin sodium clathrate API (**B**), warfarin API (**C**), and warfarin oral suspension sediment (**D**) particles.

The morphology of the three types of API particles was observed using optical microscopy. The amorphous sodium salt was irregularly shaped (Figure 7A). Warfarin crystals appeared rod-like (Figure 7C), while the clathrate sodium salt exhibited a morphology characterized by moderate regularity of elongated crystals of various dimensions (Figure 7B). The oral suspension sediment exhibited ordered assemblies of needle-like crystals (Figure 7D).

### 2.6. UV Spectroscopy of Warfarin Solutions and Supernatants

**Figure 8:**
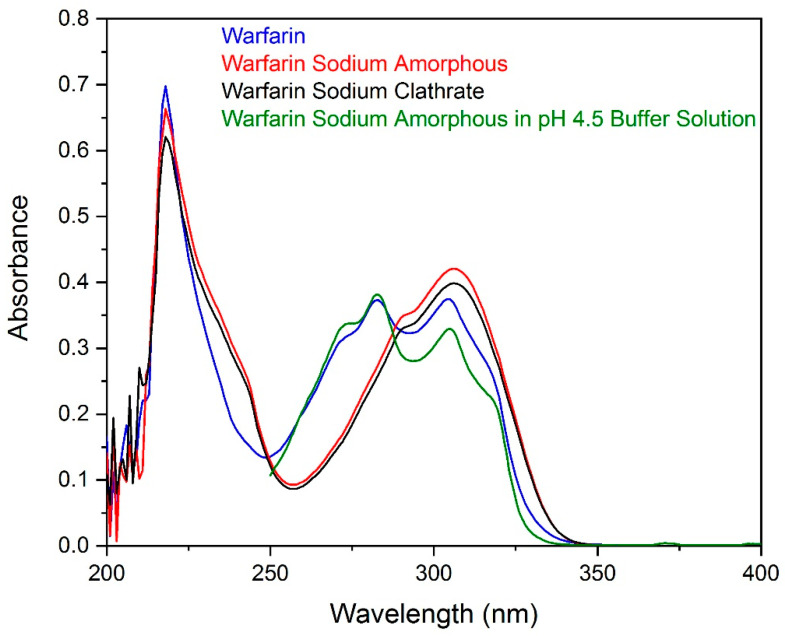
UV absorption spectra of aqueous solutions of warfarin sodium amorphous API, warfarin sodium clathrate API, warfarin API, and warfarin sodium amorphous supernatant in a buffer solution (pH = 4.5).

Aqueous solutions of the three APIs were studied using UV spectrometry. The spectra are presented in Figure 8. The pH of warfarin sodium amorphous and sodium clathrate solutions were found to be 5.6 and 5.9, respectively, while the pH of the warfarin solution was 5.1.

A quantity (Materials and Methods) of warfarin sodium in amorphous form was dissolved in buffer solution, pH = 4.5, in order to simulate the acidic conditions of the oral formulation (pH ≈ 4.5). The suspension taken after precipitation was filtrated. The spectrum of the filtrate is presented in Figure 8.

The differences in the spectra consist in the degree of ionization of warfarin, which depends on the pH of the solution, as described elsewhere [21]. A pK of 5.5 for warfarin has also been reported [22]. For the solutions with pH values near to the pK, the protonated and unprotonated warfarin ions coexist in the liquid phase. For lower values, i.e., the simulated formulation supernatant, the protonated prevails slightly. Coexistence of the sodium amorphous salt and the dried warfarin of the oral suspension supernatant was also noted in the Raman spectrum (Figure 2).

### 2.7. Dependence of Precipitation on the pH

The precipitation rate and quantity of warfarin from warfarin sodium amorphous solutions as a function of pH was examined. Buffer solutions of citric acid-Na_2_HPO_4_ (the same as for the oral formulation) were prepared in pH values ranging from 3 to 7, and 20 mg of warfarin sodium amorphous was dissolved. Dissolution was rapid in all cases. Precipitation took place immediately for pH values from 3 to 5 and more than 70% of this was recovered. For increases in pH values toward the pK, the recovery was slightly reduced and the rate of crystallization increased. In pH = 6, a solid did not precipitate until 1 day later; for pH = 7, only a small amount of solid could be collected. For these pH values that are higher than the pK, the unprotonated warfarin ion prevailed in the liquid phase and prevented precipitation due to its increased solubility.

**Figure 9:**
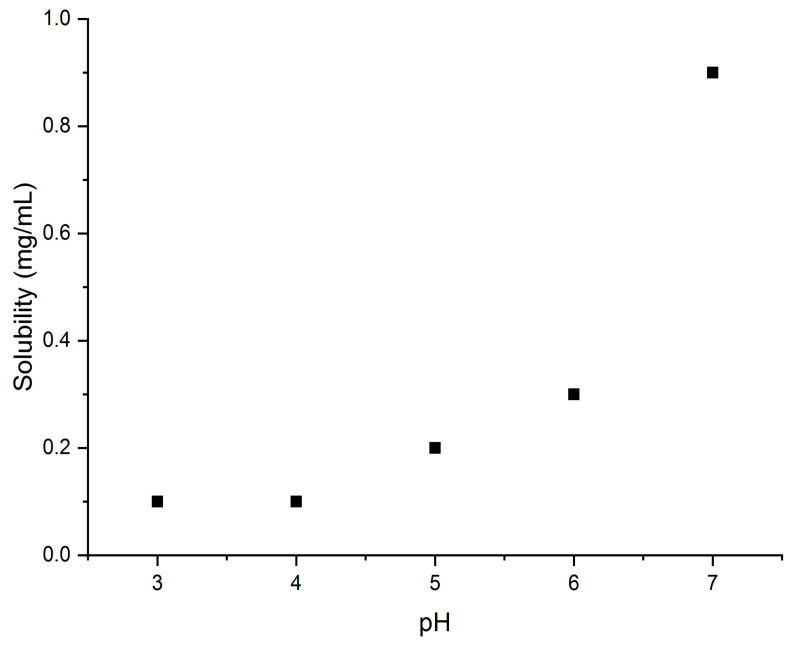
Dependance of warfarin sodium solubility on the pH value.

**Figure 10:**
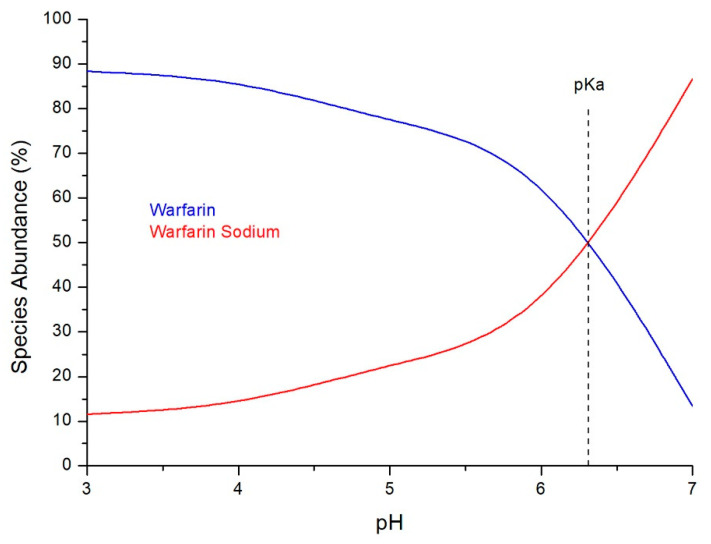
Abundance of warfarin and warfarin sodium in the pH range of 3–7.

From the amount of warfarin that was precipitated in each aqueous medium of different pH values, the solubility of warfarin sodium and the K_sp_ values were determined (Table 1). The effect of pH on the solubility of warfarin sodium is also presented in Figure 9. Furthermore, the abundance of warfarin and warfarin sodium in the pH value range of 3–7 can be found from the Bjerrum plot in Figure 10 [23].

**Figure 11:**
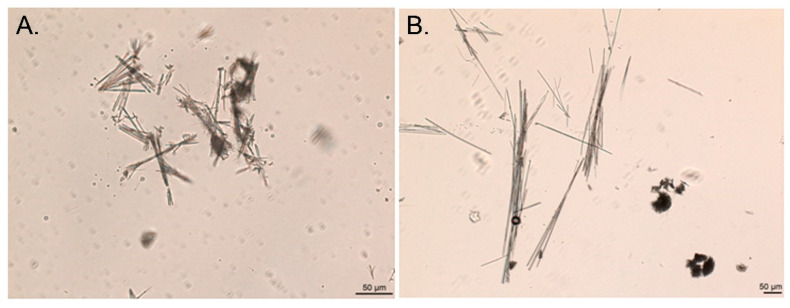
Optic microphotographs of the crystals precipitated from warfarin sodium amorphous buffer solutions at (**A**) pH = 3; and (**B**) pH = 6.

The needle-like crystal clusters of precipitate were found to comprise warfarin (Figure 11), which is in agreement with the previous results for the oral suspension.

### 2.8. Dependence of Precipitation on Temperature

The effect of temperature on the precipitation of warfarin was investigated. Aqueous solutions of warfarin sodium in amorphous form were prepared and stored at 15, 25, and 35 °C. Apart from a delay in precipitation, no other deviation was observed.

## 3. Discussion

Warfarin sodium is commercially available as an oral suspension. However, analysis of the suspension and the sediment demonstrated that the solid is the unsalted form of warfarin, which is sparingly soluble. Its limited solubility can have an effect on its bioavailability. This was the reason why the sodium form was used as API for the formulation in the first place.

The analysis of the suspension, supernatant, and sediment showed that precipitation of warfarin occurred without any intermediate stages, i.e., transformation of sodium salt to warfarin. The underlying mechanism relies on the dissociation of the sodium salt in the liquid phase and the subsequent partial protonation of the anion as the pH of the formulation was close to the pK of warfarin (5.5). The protonated form, characterized by limited solubility, precipitated as warfarin soon after dissolution of the sodium salt. The unprotonated form remained in the liquid phase as a stable undersaturated solution. The distribution of warfarin to the protonated and unprotonated entities in the supernatant was verified by UV spectroscopy and Raman spectroscopy using the dried sample.

The crystallization of warfarin from aqueous solutions of the sodium salt was found to be pH-dependent but not temperature-dependent. In more acidic pH values (less than 5), the precipitation took place immediately after API dissolution and resulted in almost 80% recovery (as warfarin) of the initial added amount (sodium salt). In order to retain solution clarity, alkaline pH values should be sought, in which warfarin ions remain unprotonated and extremely soluble.

Similar results were repeatedly found when sodium amorphous salt or sodium clathrate was dissolved in placebo solution or pure water.

Another study of the dissolution of warfarin sodium (amorphous and clathrate) tablets revealed conversion to a crystalline “acidic form” of warfarin in the acidic pH of the stomach [11]. The XRPD pattern and Raman spectrum of this form were essentially identical to those for warfarin in our work.

From an analytical point of view, chemical identification of the solid was accomplished using Raman and IR spectroscopy, X-ray diffraction, and DSC. Among them, Raman spectroscopy was the most useful as it was applicable to the suspension (as received), the sediment, and the dried supernatant. DSC was the only technique that could discriminate between all three forms. IR is suitable only for solids, whereas XRD is suitable for crystalline solids and not for the amorphous form.

## 4. Materials and Methods

### 4.1. Reagents

The commercial oral suspension used (1 mg/mL) was from Rosemont Pharmaceuticals^®^ (Rosemont Pharmaceuticals Limited, Leeds, UK). The warfarin sodium in amorphous form (Cilag AG, Schaffhausen, Switzerland), the corresponding placebo suspension, and all the excipients (both in solid and liquid form) were kindly provided by Pharmadata SA (Lavrion, Greece). Warfarin sodium clathrate (Cilag AG, Schaffhausen, Switzerland) and warfarin (Sigma Aldrich^®^, Saint Louis, MO, USA) were kindly provided by the Laboratory of Instrumental Pharmaceutical Analysis of Aristotle University, Thessaloniki, Greece. The excipients of warfarin placebo are presented in Table 2.

### 4.2. Solutions

Solutions of warfarin sodium amorphous salt and warfarin sodium clathrate in water were prepared at concentrations 1, 6, and 10 mg/mL by dispersing carefully weighed amounts of the solids into an appropriate quantity of the aforementioned liquids followed by stirring.

### 4.3. Raman Spectroscopy

A Raman spectrometer (InVia Reflex Raman spectometers, Renishaw, Wotton-under-Edge, UK) equipped with an optical microscope (Research Grade, Leica DMLM microscope) and a laser with a 785 nm excitation line was used. The laser line was focused through a 20× objective lens on the sample. The system was equipped with a CCD detector (Peltier cooled, near infrared enhanced). The power of the incident laser was 250 mW. The typical spectral resolution was 2 cm^−1^. Windows-based software was used (WiRE© 2.0) to obtain the spectra. Instrument response (laser power and the wavenumber) was checked by recording the spectrum of Si.

For Raman spectra collection, the samples were placed on a highly reflective sample carrier (EMF Corporation, Ithaca, NY, USA). Quantities of a few milligrams for the solids and a few microliters for the liquids or slurries were used. The recorded spectra were the sum of 5 scans of 20 s exposure time and the acquired region was 250–2000 cm^−1^.

### 4.4. X-ray Powder Diffraction (XRPD)

For the XRPD measurements, an X-ray powder diffractometer (Bruker AXS D2 Phaser 2nd Gen, Karlsruhe, Germany) with Bragg–Brentano geometry, a LynxEye detector, and Cu Kα spectral line (λ = 1.54184 Å) were used. The scan mode was continuous, the step size was 0.02° (2θ), and the scan speed was set at 2.0 s/step. The angles scanned ranged from 2 to 30° 2θ. The primary divergence slit was 0.6 mm, the air scatter screen was 3 mm, and voltage and current were set at 30 kV and 10 mA, respectively. The instrument performance and its accuracy were checked against the recording of corundum reference sample A26-B29-S provided by Bruker. The powders were placed into a 25 mm diameter and 1.5 mm depth circular cavity of a PMMA holder, using a glass slide. For samples of a minimum amount, an Si low background sample holder was used.

### 4.5. Optical Microscopy

An optical microscope (Leica DM 2500M, Leica Microsystems Ltd., Heerbrugg, Switzerland) equipped with a video camera (Leica DFC420 C, Leica Microsystems Ltd., Heerbrugg, Switzerland) was used to obtain pictures of the API crystals in transmittance mode and brightfield illumination, using the 10×, 20×, and 40× Leica objective lenses.

Approximately 3 mg of the powder samples were dispersed in 3 mL of mineral oil (ACRŌS ORGANICS, Geel, Belgium) while the suspensions were analyzed as received. One or two drops of the aforementioned samples were then placed on a 76 mm × 26 mm × 1 mm microscope slide (Paul Marienfeld GmbH & Co. KG, Lauda-Königshofen, Germany), covered with a 22 mm × 22 mm cover slip (Paul Marienfeld GmbH & Co. KG, Lauda-Königshofen, Germany). Windows-based software (LAS© V4.11) was used for image acquisition and analysis.

### 4.6. Differential Scanning Calorimetry (DSC)

The DSC measurements were performed with a thermal analyzer (Q100, TA Instruments, Newcastle, DE, USA). The temperature ranged from 25 to 225 °C, the heating rate was 10 °C/min, and the sample was placed in a nitrogen atmosphere during measurement.

### 4.7. IR Spectroscopy

The spectra were obtained using a FTIR-ATR (PerkinElmer Spectrum 100, Shelton, CT, USA) on a 1 bounce Diamond-ZnSe Crystal. Each spectrum was the sum of 30 scans, which were obtained in the region of 4000–650 cm^−1^, using a spectral resolution of 4 cm^−1^.

### 4.8. UV Spectroscopy

Warfarin, warfarin sodium amorphous, and warfarin sodium clathrate were dispersed in water at a concentration of 1 mg/mL. Filtration through 0.22 μm GSWP nitrocellulose membrane filters (Merck Millipore Ltd., Cork, Ireland) and a vacuum pump (KNF Neuberger Inc. Laboport, Trenton, NJ, USA) followed. Warfarin sodium amorphous and sodium clathrate filtrates were diluted 100-fold in water. Warfarin filtrate was further used as received. Purified water served as blank solution for spectra acquisition.

The UV spectra were acquired using a Cary 60 UV-Vis Spectrophotometer (Agilent Technologies, Santa Clara, CA, USA), in the wavelength range of 250–400 nm and a scan rate of 300 nm/min.

### 4.9. pH Measurements

McIlvaine buffer solutions with pH 3, 4, 5, 6, and 7 were prepared by mixing appropriate volumes of 0.1 M citric acid monohydrate and 0.2 M Na_2_HPO_4_ (Table 3) [24].

The percentage of warfarin sodium precipitated as warfarin crystals was determined by dividing the weight of the precipitate by the molecular weight of warfarin (308.3 g/mol) and multiplying by the molecular weight of warfarin sodium (330.3 g/mol). Knowing the abundance (% mass) of warfarin and warfarin sodium in each pH, a Bjerrum plot [23] can be created. The value of pKa of warfarin can be estimated from the intercept of the curves of warfarin and warfarin sodium. When warfarin sodium is dissolved in an aqueous medium, warfarin anions and sodium cations are produced in a stoichiometric ratio 1:1. The molarity of the dissolved warfarin anions, [Warfarin^−^], and sodium cations, [Na^+^], in each pH value was determined by the following equation:

(1) $$[{\mathrm{Warfarin}}^{-}]=[{\mathrm{Na}}^{+}]=\frac{m_{\mathrm{WS}}\;\left(g\right)\;\times \;1000\;\mathrm{mL}/L}{V\;\left(\mathrm{mL}\right)\;\times {\;\mathrm{MW}}_{\mathrm{WS}}}$$

where m_WS_ is the mass of warfarin sodium that remained dissolved (in grams), V the volume of the aqueous solution (in mL) and MW_WS_ the molecular weight of warfarin sodium (330.3 g/mol). The value of K_sp_ for each pH was determined by Equation (2).

K_sp_ = [Warfarin^−^] · [Na^+^] (2)

The solubility of warfarin sodium in mg/mL in each pH value was calculated by dividing the mass of the dissolved warfarin sodium by the volume of the solution. The square root of K_sp_ is also equal to the solubility (M) of warfarin sodium in the respective pH.

### 4.10. Temperature Experiments

A water bath (Valka K10-W5, VAL-Electronic, Nea Erythraia, Greece) coupled with a thermocirculator (Valka TP 100, VAL-Electronic, Nea Erythraia, Greece) was used at temperatures 15, 25, and 35 °C. Fresh solutions, in triplicates, were prepared for each temperature.

## Figures and Tables

**Table 1:** Effect of pH on the solubility of warfarin sodium and its K_sp_ values.

pH	Solubility (mg/mL)	K_sp_ (M^2^)
3	0.1	1.2 × 10^−7^
4	0.1	1.6 × 10^−7^
5	0.2	4.8 × 10^−7^
6	0.3	8.2 × 10^−7^
7	0.9	6.9 × 10^−6^

**Table 2:** Ingredients of warfarin placebo (excipients) as received from the Pharmadata SA.

Ingredients	Function
Propylene glycol (E1520) ^1^	solvent
Benzoic acid (E210)	antimicrobial agent
Xanthan gum	suspending agent
Polysorbate 80	emulsifier
Citric acid (E330)	adjusts pH
Disodium phosphate	adjusts pH
Aluminum magnesium silicate	suspending agent
Liquid maltitol 75% (E965) ^1^	solvent
Masking flavor	flavor
Purified water^1^	solvent

^1^ The three major liquid excipients of the oral suspension are purified water 66%, liquid maltitol 27%, and propylene glycol 4%.

**Table 3:** Preparation of McIlvaine buffer solutions [24].

pH	0.1 M Citric Acid (mL)	0.2 M Na_2_HPO_4_ (mL)
3	15.89	4.11
4	12.29	7.71
5	9.70	10.30
6	7.37	12.63
7	3.53	16.47

## Data Availability

Data is contained within the article.
